# Prehistoric Mining in North Carolina

**Published:** 1881-04

**Authors:** 


### PREHISTORIC MINING IN NORTH CAROLINA.

Mining for mica has become a profitable pursuit in North Carolina. It is a curious fact that the best mines are located upon sites which afford evidence of having been worked in prehistoric times, and are called there "old diggings." Most of the old works probably belonged to the mould-builders, but a tradition coming down from the Indians ascribes some of them to white men. The tradition has recently been confirmed by the discovery of old implements of iron in a prehistoric shaft in Macon County, which are fully described and figured by Mr. F. W. Simonds, in the "American Naturalist." The implements were found in the rubbish which had accumulated within the shaft, between thirty-five and fifty feet below the surface, and consist of an axe of a pattern now rarely met with, light in weight, and having on the blade a brand which has been nearly effaced by erosin; two articles which were evidently gudgeons of a windlass, with heads pronged for the insertion of levers, pointed at the ends, so that they could be driven into a wooden roller, and having the lower part of the shank squared to prevent them from turning in the wood, and the upper part round so as to serve as an axle for the roller; and a wedge with battered head. All were of wrought iron, and had probably been worn out and thrown away. Mr. Simonds suggests that they are the relics of a party of Spaniards who left one of the ancient colonies or expeditions on a "prospecting tour" and tried the mines. Less palpable evidence of more skillful mining than that of any aborigines have been found in other shafts.—*The Popular Science Monthly.*

